# Relationship of endoscopic lesions of the renal papilla with type of renal stone and 24 h urine analysis

**DOI:** 10.1186/s12894-020-00615-4

**Published:** 2020-04-25

**Authors:** X.A. Sabaté Arroyo, F. Grases Freixedas, J. L. Bauzà Quetglas, J. Guimerà Garcia, E. Pieras Ayala

**Affiliations:** 1 Hospital Universitari Son Espases, Ctra. Valldemossa, 79, Palma de Mallorca, Spain; 2 Universitat de les Illes Balears. IUNICS, Ctra. de Valldemossa, Km. 7.5, Palma de Mallorca, Spain

**Keywords:** Randall’s plaque, Tubular calcification, Papillary crater, Hypercalciuria, Hypocitraturia

## Abstract

**Background:**

Our purpose was to study the relationship of the 3 different types of endoscopic calcifications of the renal papilla (Randall’s plaque, intratubular calcification, papillary crater) with the type of stone and urine analysis.

**Methods:**

This prospective study examined 41 patients (age range: 18 to 80 years) who received retrograde intrarenal surgery (RIRS) for renal lithiasis (mean stone size: 15.3 ± 7.2 mm). The renal papilla injuries were endoscopically classified as Randall’s plaque, intratubular calcification, or papillary crater. Calculi were classified as uric acid, calcium oxalate monohydrate (COM; papillary and cavity), calcium oxalate dihydrate (COD), or calcium phosphate (CP). A 24 h urine analysis of calcium, oxalate, citrate, phosphate, and pH was performed in all patients. The relationship of each type of papillary injury with type of stone and urine chemistry was determined. Fisher’s exact test and Student’s t-test were used to determine the significance of relationships, and a *p* value below 0.05 was considered significant.

**Results:**

The most common injury was tubular calcification (78%), followed by Randall’s plaque (58%), and papillary crater (39%). There was no significant relationship of Randall’s plaque with type of stone. However, endoscopic intratubular calcification (*p* = 0.025) and papillary crater (*p* = 0.041) were more common in patients with COD and CP stones. There were also significant relationships of papillary crater with hypercalciuria (*p* = 0.036) and hyperoxaluria (*p* = 0.024), and of Randall’s plaque with hypocitraturia (*p* = 0.005).

**Conclusions:**

There are certain specific relationships between the different types of papillary calcifications that were endoscopically detected with stone chemistry and urine analysis. COD and CP stones were associated with endoscopic tubular calcifications and papillary craters. Hypercalciuria was associated with tubular calcification, and hypocitraturia was associated with Randall’s plaque.

## Background

In 1937 Alexander Randall first established a relationship between a non-inflammatory subepithelial calcification and the onset of kidney stone formation [1]. Although many subsequent studies have examined the mechanisms and causes of Randall’s plaques, the conclusions of many of these studies still require verification [2].

Papillary calculi can initiate in the papillary tissue or inside the Bellini ducts, and there are therefore two different types of stone formation. First, calculi may initiate in papillary tissue following oxidative stress, exposure to cytotoxic substances, or other factors that injure collagen-rich areas near the loop of Henle and the cubic epithelium, which covers the papilla. Because the interstitial fluid has a pH of 7.4, and is therefore always supersaturated with calcium phosphate, cellular detritus or oxidized cellular collagen can act as a heterogeneous nucleant, leading to the deposit of hydroxyapatite (HAP). If this deposit grows enough to break through the epithelium that covers the papilla, it can contact the urine. Because the urine is always supersaturated with calcium oxalate, a calcium oxalate monohydrate (COM) calculus will form when there is a deficiency of crystallization inhibitors and/or an alteration of the immune system. Stones can also form when there is intratubular injury of the Bellini ducts and urine supersaturation, in which there are large amounts of calcium oxalate dihydrate (COD) and/or HAP crystals. In this case, obstruction of the collecting tubules and deposits that form in the most distal part of the tubule, upon contacting the urine, generate the renal calculi [3–5].

Obviously, not all kidney stones develop by processes that involve the renal papillae. Thus, the presence of morpho-anatomical factors, such as cavities that have low urodynamic efficacy and anatomical malformations, can lead to urine stasis, the accumulation of stagnant urine, and stone formation, as also occurs in the urinary bladder. The renal papillae have no role in the formation of these stones [5–7].

Because of advances in urological technologies and endoscopy, and the increasing comfort and dexterity of the surgeons in performing endoscopic procedures, retrograde intrarenal surgery (RIRS) has become a common method for treatment of renal stones. These techniques of renal exploration allow better treatment of renal stones and more thorough examination of the renal papillae [8, 9]. Although endoscopic renal surgery can detect a wide range of injuries to the renal papilla surface, the relationships of these different injuries with the etiopathogenic mechanisms of renal stone formation are unclear [10].

The purpose of this paper is to study the relationship between injuries detected endoscopically of the renal papilla detected by endoscopy with the type of renal calculus and 24 h urine analysis.

## Methods

Forty-one consecutive patients with renal lithiasis and indications for RIRS were recruited prospectively from our institution. All patients provided informed consent for participation in the study, and the study was approved by the ethics committee of our hospital (No. 3105/15P, Comité de Ética de la Investigación de las Islas Baleares- CEI-IB). All research was performed in accordance with relevant guidelines/regulations.

**Fig. 1:**
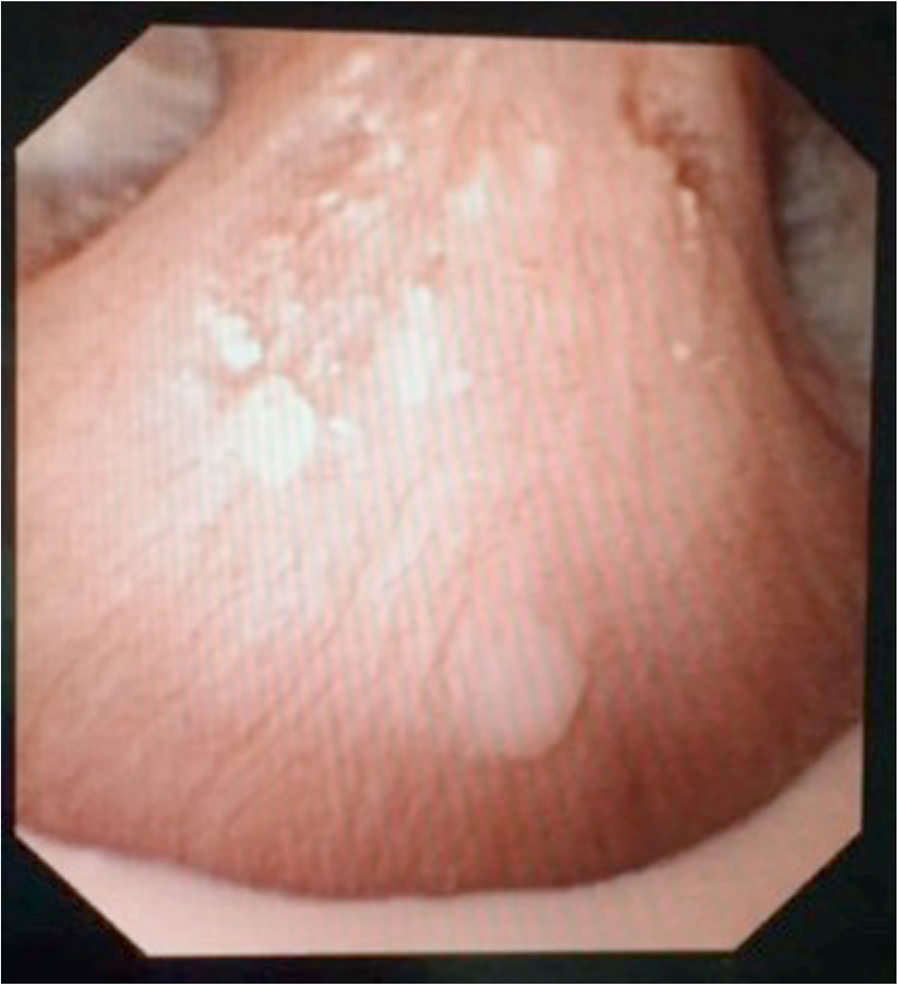
Example of Randall’s plaque

**Fig. 2:**
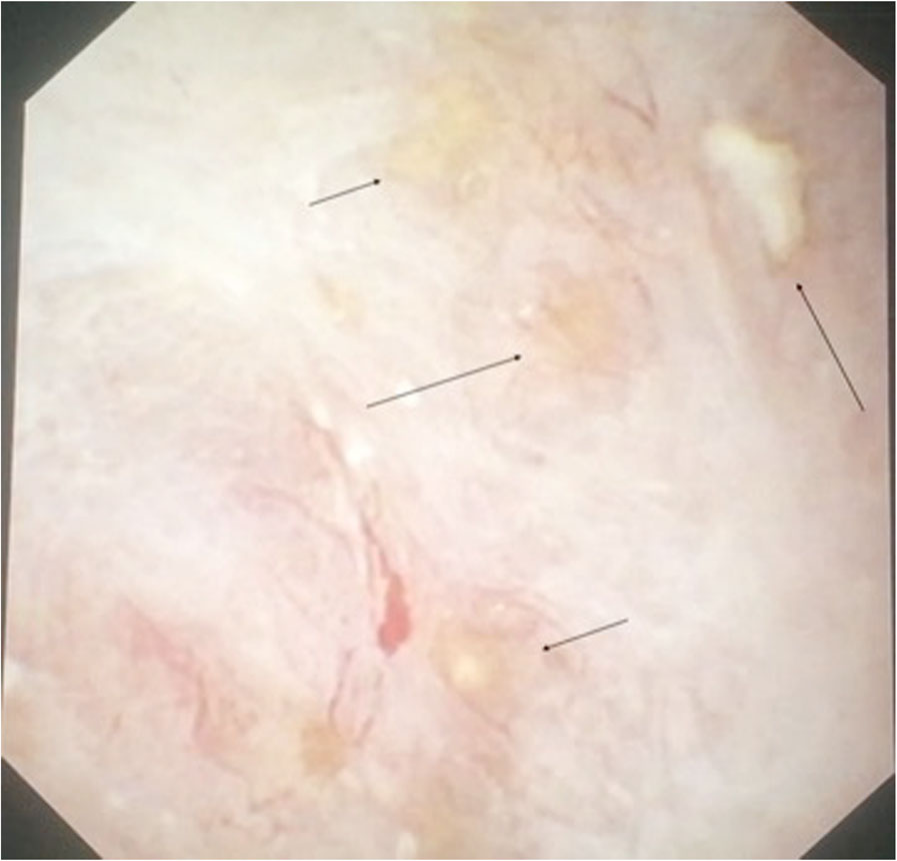
Example of Intratubular calcifications

**Fig. 3:**
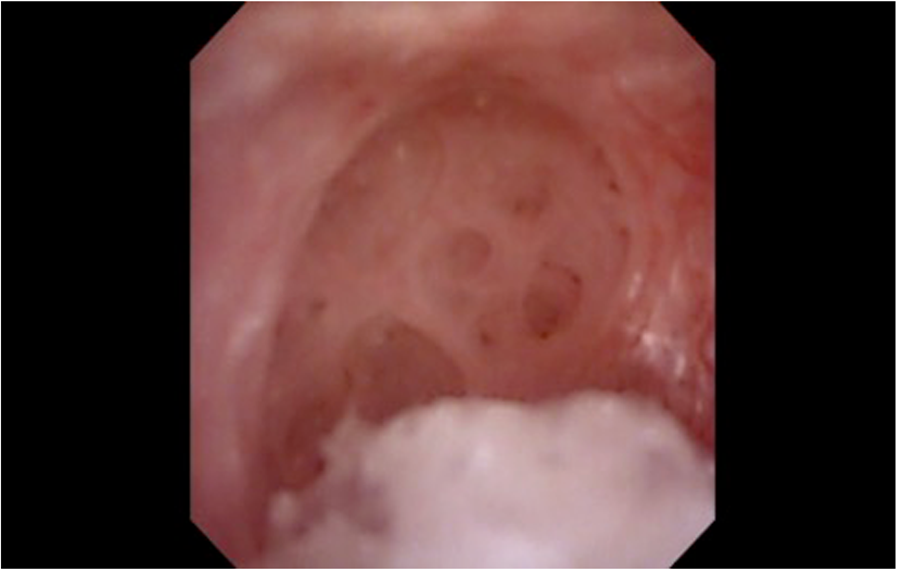
Example of Papillary cráter

We measured stone size using computed tomography prior to surgery. Systematic endoscopic examination of the renal papilla was performed using a flexible digital ureteroscope (Olympus URF-V and Karl Storz Flex Xc). The procedure started with examination of the upper calyx, ended in the lower calyx, and photographs were taken in the area of the renal papillae. The 3 types of injury in the papilla were defined as previously described [11]:

- Randall’s plaque (Fig. 1): White deposits of intrarenal HAP calcifications, near the epithelium of the papilla, with irregular shape.
- Intratubular calcification or Randall’s plug (Fig. 2): Yellow punctate deposit on the surface of the renal papilla or at the end of a dilated terminal collection tubule.
- Papillary crater (Fig. 3): Slit-shaped injury or crater in the surface of a renal papilla.

After laser lithotripsy, some stone fragments were extracted to determine calculus type. In particular, electron microscopy and infrared spectroscopy were used to classify stones into 1 of 4 groups: calcium oxalate monohydrate (COM; papillary and cavity), calcium oxalate dihydrate (COD), uric acid, and calcium phosphate (HAP and mixed stones) [12, 13]. Struvite and brushite stones were not considered because their mechanisms of formation are completely different.

At 30 to 60 days after surgery, 24 h urinary chemistry was analyzed in each patient. The results were used to classify patients as having hypercalciuria (> 220 mg/24 h or > 17 mg/dL), hyperoxaluria (> 40 mg/24 h or > 3 mg/dL), hypocitraturia (< 350 mg/24 h or < 23 mg/dL), or hyperphosphaturia (> 1100 mg/24 h or > 100 mg/dL), as previously described [12–14].

The relationships of each endoscopic papillary injury with type of stone, urinary biochemical parameters, and urinary pH were determined. Each value is reported as mean ± standard deviation. Fisher’s exact test was used for the analysis of qualitative variables, and the Student’s *t*-test for the comparison of means. A *p* value below 0.05 was considered significant.

## Results

The average patient age was 48 years (±13), and 54% of the patients were male. A total of 58% of the patients had Randall’s plaque, 78% had intratubular calcification, and 39% had a papillary crater. Thus, 54% of the patients had 2 or more different types of renal injury, and 46% only had one kind of renal injury.

The stone mean size (i.e., largest diameter) was 15.4 mm (±7.4). Urinary biochemistry indicated the mean pH was 6.0 (±0.79), mean 24 h urine volume was 1863 mL (±901), mean calcium level was 218 mg/24 h (±127), mean phosphate level was 835 mg/24 h (±312), mean oxalate level was 27 mg/24 h (±12), and mean citrate level was 398 mg/24 h (±300).

There was no significant relationship of Randall’s plaque with the type of stone (*p* = 0,39) (Table 1). However, there were significant relationships of endoscopic intratubular calcification (*p* = 0.025) and papillary crater (*p* = 0.041) with type of stone, in that COD and calcium phosphate stones were more common in both groups (Tables 2 and 3).

**Table 1:** Randall's Plaque

	RANDALL’S PLAQUE		
	YES	NO	Significance
**Patients (*N*** **= 41)**	**24 (59%)**	**17 (40%)**	
**COM**	**5 (71%)**	**2 (28%)**	**0,390**
**COD**	**7 (77%)**	**2 (22%)**	
**Uric Acid**	**3 (50%)**	**3 (50%)**	
**Calcium Phosphate**	**9 (47%)**	**10 (52%)**	
**pH**	**6,0 ± 0,81**	**6,19 ± 0,78**	**0,48**
**Diuresis (mL)**	**1965 ± 966**	**1580 ± 487**	**0,56**
**Calcium (mg/24 h)**	**202,09 ± 119,78**	**247,0 ± 140,53**	**0,37**
**Oxalate (mg/24 h)**	**28,35 ± 13,54**	**26,77 ± 9,32**	**0,72**
**Citratoe (mg/24 h)**	**391,77 ± 326,12**	**409,69 ± 260,37**	**0,86**
**Calcium (mg/dL)**	**11,21 ± 7,11**	**15,76 ± 10,13**	**0,09**
**Phosphate (mg/dL)**	**52,60 ± 30,31**	**59,21 ± 32,59**	**0,62**
**Citrate (mg/dL)**	**21,35 ± 19,90**	**28,37 ± 14,82**	0,005*
**Oxalate (mg/dL)**	**1,83 ± 1,19**	**1,79 ± 0,91**	**0,98**
**Uric acid (mg/dL)**	**30,07 ± 16,91**	**39,31 ± 21,69**	**0,16**

**Table 2:** Tubular calcification

	TUBULAR CALCIFICATION		
	YES	NO	Significance
**Patients (*N*** **= 41)**	**32 (78%)**	**9 (21%)**	
**COM**	**3 (42%)**	**4 (57%)**	0,02*
**COD**	**9 (100%)**	**0**	
**Uric Acid**	**4 (66%)**	**2 (33%)**	
**Calcium Phosphate**	**16 (84%)**	**3 (15%)**	
**pH**	**6,1 ± 0,75**	**5,6 ± 0,89**	**0,12**
**Diuresis (mL)**	**1990 ± 963**	**1619 ± 742**	**0,67**
**Calcium (mg/24 h)**	**234,21 ± 123,98**	**162,63 ± 132,29**	**0,16**
**Oxalate (mg/24 h)**	**28,52 ± 11,6**	**25,19 ± 14,05**	**0,49**
**Citrate (mg/24 h)**	**416,29 ± 283,35**	**335,08 ± 367,86**	**0,50**
**Calcium (mg/dL)**	**14,15 ± 9,51**	**9,68 ± 3,67**	0,036*
**Phosphate (mg/dL)**	**55,23 ± 31,29**	**56,01 ± 32,08**	**0,49**
**Citrate (mg/dL)**	**23,60 ± 16,41**	**26,86 ± 23,87**	**0,42**
**Oxalate (mg/dL)**	**1,74 ± 1,07**	**2,06 ± 1,07**	**0,67**
**Uric acid (mg/dL)**	**34,27 ± 19,91**	**33,06 ± 18,55**	**0,90**

**Table 3:** Papillary crater

	PAPILLARY CRATER		
	YES	NO	Significance
**Patients (*N*** **= 41)**	**16 (39%)**	**25 (61%)**	
**COM**	**1 (14%)**	**6 (85%)**	0,04*
**COD**	**7 (77%)**	**2 (22%)**	
**Uric Acid**	**2 (33%)**	**4 (66%)**	
**Calcium Phosphate**	**6 (31%)**	**13 (68%)**	
**pH**	**6,22 ± 0,82)**	**5,97 ± 0,77**	**0,33**
**Diuresis (mL)**	**2328 ± 1092**	**1560 ± 603**	**0,45**
**Calcium (mg/24 h)**	**265,87 ± 128,55**	**184,33 ± 118,27**	0,05*
**Oxalate (mg/24 h)**	**28,79 ± 12,84**	**27,05 ± 11,75**	**0,67**
**Citrate (mg/24 h)**	**501,80 ± 363,51**	**324,27 ± 226,96**	**0,08**
**Calcium (mg/dL)**	**13,12 ± 10,31**	**13,17 ± 7,82**	**0,52**
**Phosphate (mg/dL)**	**51,31 ± 40,24**	**57,86 ± 24,64**	**0,58**
**Citrate (mg/dL)**	**25,96 ± 20,77**	**23,36 ± 16,59**	**0,41**
**Oxalate (mg/dL)**	**1,33 ± 0,69**	**2,11 ± 1,19**	0,024*
**Uric Acid (mg/dL)**	**30,21 ± 23,74**	**36,27 ± 16,35**	**0,58**

There were also significant relationships between papillary crater and hypercalciuria (*p* = 0.036) and hyperoxaluria (*p* = 0.024), and between Randall’s plaque and hypocitraturia (*p* = 0.005). None of the other relationships were statistically significant.

## Discussion

Our results suggest that the type of papillary injury identified by endoscopy is associated with the type of stone and with certain disorders in urinary analysis. This suggests that different mechanisms are responsible for these different endoscopic papillary injuries.

Coe et al. [15] hypothesized that the direct contact of a plug blocking Bellini’s duct in an environment with hypercalciuric urine favors the formation of HAP and calcium oxalate stones, although this relationship has not been demonstrated previously. Our results are consistent with this hypothesis. In particular, we observed that intratubular calcification and papillary crater each had significant associations with the type of lithiasis, in that they were more prevalent in patients with COD and CP stones. Moreover, we found that Randall’s Plaque was mainly associated with COM calculi.

We also found that intratubular calcification was significantly associated with urinary calcium concentration. This result confirms that Bellini’s duct must be exposed to supersaturated urine for the formation of HAP stones (pH > 6) and to hypercalciuria for the formation of an intratubular obstruction. When this deposit reaches the end of Bellini’s duct and contacts the urine, it can induce the development of COD or COD+HAP calculi.

On the other hand, Randall’s plaque was mainly associated with COM calculi that developed in renal cavities. It is important to consider that typical calculi of papillary COM are generated on Randall’s plaque, which originates from internal lesions of intrapapillar tissue [6]. These are small stones that are usually expelled spontaneously. Consequently, this type of calculus does not require surgery. However, all the stones examined in the present study required RIRS for elimination because they were larger, developed on the most distal part of Bellini’s ducts, and had fast growth due to the existence of hypercalciuria and sometimes high urinary pH. Therefore, we expected that none of the included patients had typical COM papillary calculi. We did observe the presence of Randall’s plaques, which in principle are related to the formation of COM stones. However, recent studies showed that not all of Randall’s plaques generate papillary COM calculi [6].

In fact, Randall’s plaque seems to occur without symptoms in most individuals, and stone formation only occurs when there are large numbers of these plaques [6]. It is thus possible that the small number of Randall’s plaque lesions and their total area in these patients were insufficient to lead to the development of papillary COM stones. We did not evaluate the percentage of surface affected by Randall’s plaque. However, other studies reported that the surface affected by Randall’s plaque was 7.4% in those who developed papillary calculi, but only 0.5% in those who did not develop calculi [16–22].

We found that Randall’s plaque was significantly associated with hypocitraturia (*p* = 0.005) and that urinary calcium concentration was significantly lower than those detected in the presence of intratubular calcification and / or renal crater. These data are consistent with the current understanding that a deficiency of stone inhibitors and normocalciuria can lead to the development of COM calculi [6]. Thus, citrate inhibits the crystallization of oxalate and calcium phosphate, preventing the formation of the lithiasic core and posterior growth. Interestingly, regardless of the type of injury, the presence of uric acid stones is more likely when the urinary pH is below 5.5, and the presence of HAP stones is more likely when the urinary pH is above 6.0 [23–29].

**Fig. 4:**
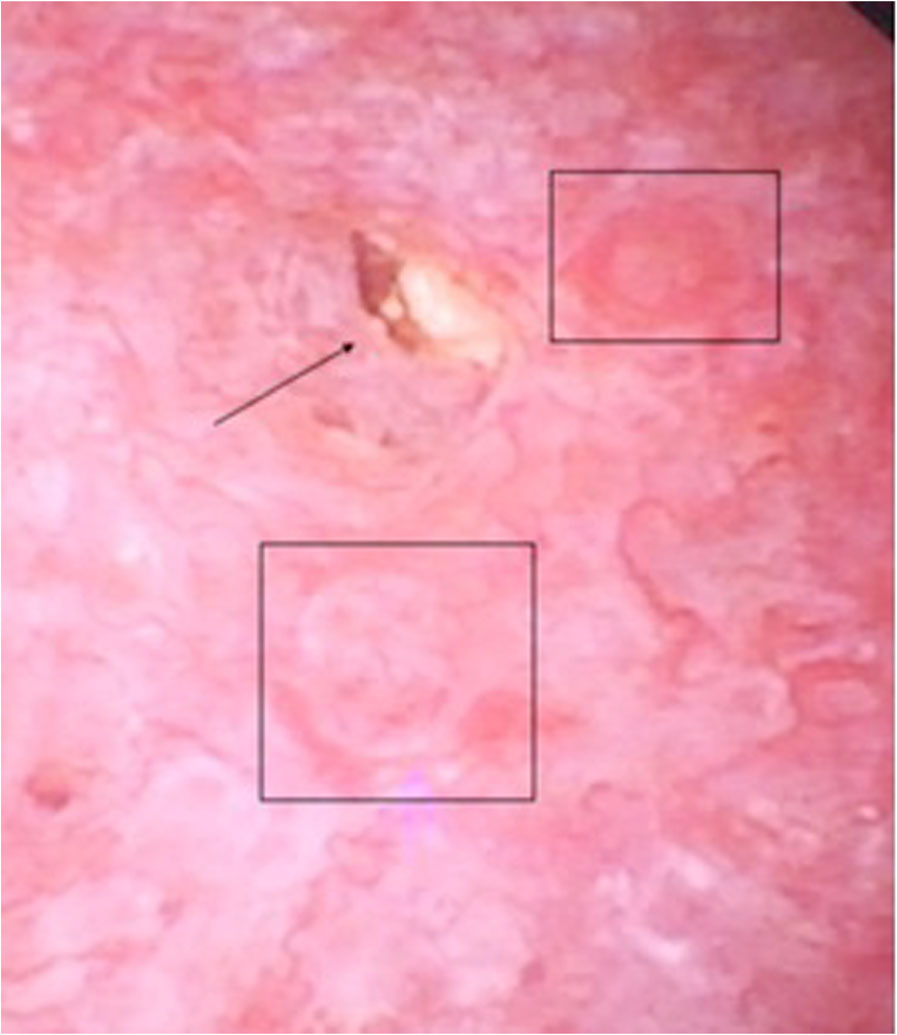
Intratubular calcification and Papillary crater in same papilla

There were some limitations in our study. First, we only described the experience of 41 patients at a single center. Second, the identification of papillary injury was somewhat subjective, even though we used previously described criteria to define the different types of injuries. Third, we did not consider the severity of papillary injuries, and only considered the presence or absence of a lesion. Another possible limitation is that there may have been some overlap in the different types of lesions if they were at different stages of disease progression. For example, we found an association of intratubular calcification with papillary crater. This can occur if lithiasis begins in an intratubular calcification, but detachment of the calculus from the renal papilla leads to the formation of a papillary crater (Fig. 4). The last limitation of the study is that the urine analysis was done at the 30–60 day after surgery and this could make changes in the urine chemistry.

Our study identified several significant relationships of endoscopic papillary injuries with the type of stone and urine chemistry. The characterization of a papillary endoscopic injury may help a urologist to better understand the underlying mechanism of stone formation in individual patients. Further studies with larger numbers of patients are needed to confirm this hypothesis.

## Conclusions

We identified certain specific relationships between different endoscopic types of papillary calcifications with stone and urine chemistry. In particular, COD and CP stones are associated with endoscopic tubular calcifications and papillary craters; hypercalciuria is associated with endoscopic tubular calcification; and COM calculi, hypocitraturia, and normocalciuria are associated with Randall’s plaque.

## Data Availability

The datasets used and/or analysed during the current study available from the corresponding author on reasonable request.

## Abbreviations

UA: Uric Acid
COM: Calcium oxalate monohydrate
COD: Calcium oxalate dihydrate
CP: Calcium phosphate
